# Independent verification system for intracavitary brachytherapy based on a reference plan and statistical model

**DOI:** 10.1093/jrr/rraf007

**Published:** 2025-03-05

**Authors:** Yuichi Akino, Fumiaki Isohashi, Takehiro Arimura, Shinichi Inoue, Hiroya Shiomi, Kazuhiko Hayashi, Shotaro Tatekawa, Keisuke Tamari, Takero Hirata, Masaki Nakai, Shinichi Shimizu, Kazuhiko Ogawa

**Affiliations:** Department of Radiation Oncology, Osaka University Graduate School of Medicine, 2-2 Yamadaoka, Suita, Osaka 565-0871, Japan; Department of Radiation Oncology, Osaka University Graduate School of Medicine, 2-2 Yamadaoka, Suita, Osaka 565-0871, Japan; Department of Radiation Oncology, Nara Medical University, 840 Shijo-Cho, Kashihara, Nara 634-8521, Japan; Department of Medical Technology, Osaka University Hospital, 2-15 Yamadaoka, Suita, Osaka, Japan; Department of Medical Technology, Osaka University Hospital, 2-15 Yamadaoka, Suita, Osaka, Japan; Department of Radiation Oncology, Osaka University Graduate School of Medicine, 2-2 Yamadaoka, Suita, Osaka 565-0871, Japan; Department of Radiation Oncology, Osaka University Graduate School of Medicine, 2-2 Yamadaoka, Suita, Osaka 565-0871, Japan; Department of Radiation Oncology, Osaka University Graduate School of Medicine, 2-2 Yamadaoka, Suita, Osaka 565-0871, Japan; Department of Radiation Oncology, Osaka University Graduate School of Medicine, 2-2 Yamadaoka, Suita, Osaka 565-0871, Japan; Department of Radiation Oncology, Osaka University Graduate School of Medicine, 2-2 Yamadaoka, Suita, Osaka 565-0871, Japan; Department of Radiation Oncology, Osaka University Graduate School of Medicine, 2-2 Yamadaoka, Suita, Osaka 565-0871, Japan; Department of Carbon Ion Radiotherapy, Osaka University Graduate School of Medicine, 2-2 Yamadaoka, Suita, Osaka 565-0871, Japan; Department of Radiation Oncology, Osaka University Graduate School of Medicine, 2-2 Yamadaoka, Suita, Osaka 565-0871, Japan

**Keywords:** intracavitary brachytherapy, cervical cancer, verification system

## Abstract

High dose rate (HDR) intracavitary brachytherapy (ICBT) with a remote afterloading system plays a vital role in the treatment of cervical cancer. We aimed to develop a new verification system for ICBT for cervical cancer and evaluate the feasibility for clinical plans (Plan_Clin_) generated for different remote afterloaders, applicators and treatment techniques. In total, 517 Plans_Clin_ of patients were treated with Elekta ^192^Ir microSelectron HDR v2r. Reference plans (Plan_Ref_) were generated for the ICBT applicators. An equation to predict total dwell time (T_dwell_) of Plan_Clin_ was generated by evaluating the relationship between the volume receiving 100% of the prescribed dose (V_100%_) and the T_dwell_. We also developed software to detect human errors in Plan_Clin_ by comparing parameters, including applicator and reference point geometries, dwell position and weight patterns and reference point dose, with those of Plan_Ref_. Feasibility was evaluated for 83 Plan_Clin_ cases treated with the Elekta Flexitron remote afterloader and six ICBT plans with extra catheters (hybrid BT). The linear fitting function showed good agreement with the correlation between V_100%_ and T_dwell_. The developed equation accurately estimated the T_dwell_ of the Plan_Clin_ treated with the Flexitron with an accuracy of 0.26 ± 0.49%. Our system successfully detected intentional human errors including incorrect channel mapping, applicator tip offset, incorrect plan templates, an applicator digitization model and incorrect reference points. A verification system based on Plan_Ref_ and a statistical approach is feasible for the new remote afterloaders, applicators and hybrid BT techniques. This system contributes to the implementation of safe treatments.

## INTRODUCTION

High dose rate (HDR) intracavitary brachytherapy (ICBT) with a remote afterloading system plays an important role in the treatment of cervical cancer [1]. In the last decade, three-dimensional image-guided brachytherapy (3D-IGBT) based on computed tomography (CT) or magnetic resonance imaging (MRI) has enabled the precise localization of targets and organs at risk (OARs), accurate dose calculation and optimization of treatment plans [2]. Additionally, a hybrid of intracavitary and interstitial techniques using extra catheters or needles (hybrid BT) have shown great potential for the improvement of target coverage [3].

ICBT for cervical cancer consists of the following steps: (i) applicator insertion, (ii) CT (or orthogonal X-ray) image acquisition, (iii) treatment planning, (iv) plan verification and (v) irradiation. These steps are accomplished within a few hours. Unfortunately, a multistep ICBT procedure, which must be performed over a short period of time, can lead to errors that cause serious problems owing to the high fractional dose [4–7].

A previous analysis of incidents in external beam radiotherapy (EBRT) reported that the pre-treatment plan review by physicists was the most effective for quality control, but the effectiveness was only 63% [8]. Although not as common as for EBRT, some independent verification systems for brachytherapy are based on point dose [9–11] or 3D dose distribution [12–14]. However, many previously reported errors in brachytherapy have been attributed to human errors rather than computer calculation failures [4, 6]. The American Association of Physicists in Medicine (AAPM) task group 59 (TG-59) [15] suggested that an effective physics review encompasses much more than the verification of computer plan accuracy. Usually, a pre-treatment plan check is performed manually by a planner and/or physicists. The accuracy of the plan depends on the expertise, concentration and time available to the reviewer. In addition, the inter-institutional variability of the treatment, including applicators and planning techniques, makes standardized independent verification difficult.

Serious incidents can occur, especially when new treatment devices, applicators or techniques, such as hybrid BT, are introduced. The purpose of this study was twofold: first, to develop a simple verification method for clinical ICBT plans (Plan_Clin_), which is independent of the inter-institutional variability in remote afterloaders, applicators and planning techniques; and second, to develop a system that detects human error during treatment planning and verifies treatment data after transfer to a remote afterloader.

## METHODS

### Patients and treatment plans

**Fig. 1:**
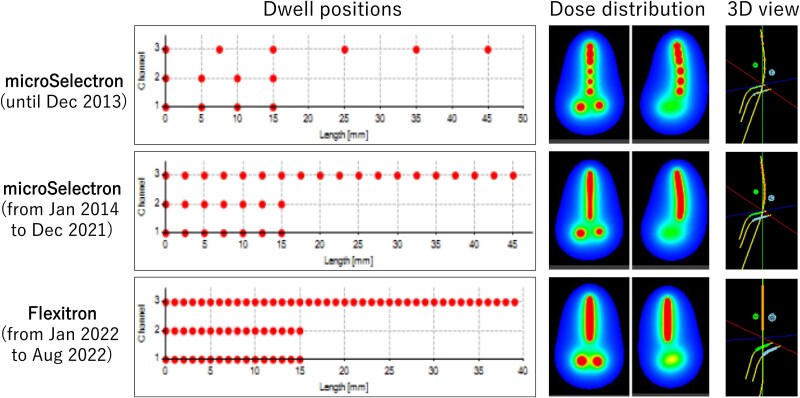
Example patterns of the dwell positions and dose distributions of 5-cm tandem and ovoid plans treated with microSelectron and Flexitron. In 3D view, two spheres represent Point A. Channel 1, 2 and 3 represent right ovoid, left ovoid and tandem applicators, respectively.

With the approval of the institutional review board, patients who underwent 3D-IGBT between April 2012 and August 2022 were retrospectively analyzed. During this period, the remote afterloader was updated. Additionally, the treatment procedure was modified. Therefore, patients were divided into three groups according to the treatment method. The number of plans for the applicator types and the use of graphical optimization are listed in Tables 1 and 2. Example patterns of the dwell positions are shown in Fig. 1. Treatment plans were generated using the Oncentra MasterPlan (Elekta) treatment planning system (TPS).

**Table 1:** Number of tandem-ovoid plans for various treatment types

	microSelectron	Flexitron
Tandem length	N	N
3 cm	3	3
4 cm	22	7
5 cm	225	28
6 cm	232	25
7 cm	20	1
Tandem curve	N	N
Small	231	
Middle	105	
Large	166	
Ovoid size	N	N
SS	231	
S	271	
Tandem orientation	N	N
Anterior	377	56
Retro	125	8
Weight optimized	N	N
None	231	6
Rescaled	105	12
Graphical	166	46
Total	N	N
	502	64

**Table 2:** Number of tandem cylinder or cylinder plans for various treatment types

	microSelectron	Flexitron
Tandem length	N	N
None	0	3
4 cm	7	7
6 cm	8	9
Cylinder diameter	N	N
2.5 cm	7	3
3.0 cm	8	16
Optimization	N	N
None	9	10
Graphical	6	2
Extra catheters	0	7
Total	N	N
	15	19

The tandem-ovoid (TO) and tandem-cylinder (TC) treatments were combined with external beam radiotherapy (EBRT). The dose of the whole pelvic radiotherapy was 30 Gy or 40 Gy, depending on the initial tumor size. The pelvis was then irradiated with a 4 cm wide central shield. The total dose delivered to the pelvic sidewall was 50 Gy/25 fractions. Details of the EBRT are described elsewhere [16].

#### microSelectron HDR

We analyzed 517 treatment plans of 155 patients treated with the microSelectron HDR v2r (Elekta, Stockholm, Sweden) between April 2012 and May 2018. Patients were treated with the Fletcher-Williamson Asia-Pacific Applicator TO and TC sets. The mean ± standard deviation of the prescribed dose per fraction for the TO plans was 6.67 ± 0.39 Gy (range 5.00–7.50 Gy). In the TC plans, the prescribed dose per fraction at Point A was 5.1 Gy.

In January 2014, the dwell position was changed from discrete to continuous. Patients treated with a vaginal cylinder without a tandem applicator were excluded because all the cylinder plans used the same patterns of weight, dwell position and dose at the reference points (Fig. 1).

#### Flexitron

In January 2022, the microSelectron HDR v2r treatment unit was replaced with the Flexitron (Elekta). The interval between each dwell point was changed from 2.5 mm to 1 mm. The patients were treated with the CT/MR Advanced Gynecological Applicator (Elekta) TO and TC sets. The tandem shape was straight (Fig. 1). The offset values for the first dwell position from the applicator tip differed from those of the old applicators.

To investigate the flexibility of the system developed in this study, 83 plans for 29 patients treated with the Flexitron between January and August 2022 were analyzed. Three plans were generated for one case using a cylinder without a tandem applicator. For two-cylinder plans and five TC plans for two patients, three or four additional catheters were inserted into the cylinder to improve the target coverage.

#### Treatment planning

Treatment plans were generated using the Oncentra MasterPlan (Elekta) TPS. The applicators were identified on the CT images using the connector-end digitization mode. The offset of the first dwell position from the applicator tip was determined by measuring it on a film. The plans were normalized at Point A as defined in the International Commission on Radiation Units and Measurements (ICRU) Report 38 [17].

A dose-volume histogram (DVH) was evaluated. For high-risk clinical target volumes (HR-CTV), the goal is ≥600 cGy/fraction delivered to 90% of the volume (D_90%_). For the OARs, our goal was to deliver <700 cGy/fraction to 2 cm^3^ (D_2 cc_). As recommended by ICRU Report 89, D_98%_ to HR-CTV and D_0.1 cc_ to OARs were also recorded. The bladder, rectum, sigmoid colon and peritoneum, representing the small intestine, were delineated as the OARs. When the OAR dose exceeded the tolerance and the D_90%_ of the HR-CTV was much higher than the goal, the Point A dose was scaled down to meet the tolerance of the OARs. If the rescaling of Point A was not sufficient to reach our goals, a graphical optimization was performed to optimize the dwell time at each dwell position.

### Plan verification software

To evaluate the validity of the plans, software was developed using Microsoft Visual Studio (C# for graphic interface, C++ for internal programs and Python for analysis of PDF files).

#### Dose calculations

The software calculated the dose using the dwell time at each point recorded in the DICOM RT-Plan file and air kerma strength (*S_k_*) registered in the verification software, compensating for source decay. A line-source model of the AAPM TG-43 equation was used [18–21]. To investigate the dose calculation accuracy of the system, the 3D dose distribution was calculated for 10 plans of three patients treated with microSelectron HDR and compared with the dose distribution exported from the Oncentra TPS as DICOM RT-Dose files. For volumes receiving doses in the range of 20% to 200% of the prescribed dose, 99% of the voxels showed a difference of ≤0.63%, indicating sufficient accuracy for calculation of clinically important parameters such as D_90%_ to the HR-CTV and D_2 cc_ to the OARs.

#### Estimation of total dwell time based on the reference plans

The TO and TC applicators were constructed by a radiation oncologist as an ideal geometry, and reference plans (Plan_Ref_) were generated on the CT images using the planning protocol at our institution. The details of generating the Plan_Ref_ were reported by Takahashi *et al.* [22], including a method to estimate the total dwell time (T_dwell_) of the ICBT based on Plan_Ref_. This method simply estimates the T_dwell_ of Plan_Clin_ from that of Plan_Ref_ corrected for *S_k_* and prescribed dose (D_presc_):


(1) \begin{equation*} {T}_{dwell, clin}=\frac{D_{presc, clin}\bullet{S}_{k, ref}}{D_{presc, ref}\bullet{S}_{k, clin}}\bullet{T}_{dwell, ref}. \end{equation*}

However, for the weight-optimized 3D-IGBT and hybrid BT plans, large errors were expected because all Plan_Ref_ are generated according to the classical Manchester system. Das *et al.* also reported an approximation formula to estimate T_dwell_ from V_100%_. Their method was not restricted to cervical cancer treatment [23]. However, the constant of their formula varied depending on the number of applicators. In this study, we developed a new approximation formula using a statistical model to accurately estimate the T_dwell_ of many types of applicators, machines and techniques. The system analyzes the DICOM CT, RT-Structure and RT-Plan files of Plan_Clin_ exported from the TPS. The 3D dose distribution within the HR-CTV and all OARs was calculated using the line-source model of the AAPM TG-43 equation [18–21] to analyze the DVH of the structures. The dose of the i^th^ voxel (D*i*) exposed from a total of *n* dwell positions was calculated as follows:


(2) \begin{equation*} {DR}_{i,j}={S}_k\bullet \varLambda \bullet \frac{G_L\left(r,\theta \right)}{G_L\left({r}_0,{\theta}_0\right)}\bullet{G}_L\bullet F\left(r,\theta \right), \end{equation*}

(3) \begin{equation*}\quad{D}_i=\sum_{j=1}^n\left({DR}_{i,j}\bullet{T}_j\right),\ \end{equation*}

where DR*_i,j_* represents the dose rate of the radiation exposed from the j^th^ dwell position with the dwell time of T*_j_*. Λ, G_L_/G_L0_, g_L_, and *F* represent the dose rate constant, geometric factor, radial dose function and anisotropy function, respectively. For HR-CTV and OARs, the dose was calculated for all voxels within the contours. For other unspecified tissues, the dose of the voxels within 30 mm of all dwell positions was calculated to evaluate the volume receiving 100% of the prescribed dose (V_100%_). For the calculation of each voxel dose, the contribution of the dwell positions within 100 mm of the voxel was considered because of the maximum range of the g_L_, and *F* values provided in the literature [20, 21]. The remaining region was ignored to accelerate the calculations. The 3D dose distribution of Plan_Ref_ was also calculated, and the relationship between T_dwell_ and V_100%_ was analyzed to obtain an equation to estimate the T_dwell_ of Plan_Clin_ from Plan_Ref_.

### Detection of human errors

The software also evaluated the validity of Plan_Clin_ by comparing multiple parameters, including the applicator and reference point geometries, dwell position, weight patterns and reference point dose, to those of Plan_Ref_. Supplementary Fig. S1 shows an example case analyzed using the verification software. The parameters analyzed using the software are listed in Table 3 and also described in Supplementary material S1 and Figs S2–4.

**Table 3:** Plan check parameters

Category	Parameters
Imaging	Elapsed time from image acquisition
	Patient position
	Device and sequence
	FOV and resolution
Plan	Plan code
	Reference plan type
	Prescribed dose
Source	Plan and current date/time
	Plan *S_k_* and calculated *S_k_*
Applicators	Number of applicators
	Dwell positions and weights
	Digitization mode
	Channel mapping
	Geometric parameters
	3D graphic view
Reference points	Number of the points
	Coordinates
	Point dose (Plan vs TG-43 calculation)
	Point dose (Plan vs reference)
	Dwell time (Plan vs estimation)
	V_100%_ (Plan vs reference)
DVH	HR-CTV D_90%_
	OARs D_2 cc_
Pre- and post-treatment	Patient ID
summaries	Plan name and plan code
	Dwell position and time

To evaluate the error detection capability of the system, we generated plans with intentional errors: (i) incorrect applicator channel mapping in TO plans, (ii) incorrect offset values for the first dwell position from the applicator tip (zero or positive/negative mistake), (iii) selection of the incorrect plan template from the library (longer or shorter tandem length), (iv) identification of the applicator coordinates with incorrect digitization mode (connector-end/tip-end), (v) incorrect reference points (incorrect coordinate and number of points) and (vi) irregular/unintended dwell points far from the target (a dwell point with was added on the tandem at 5 cm proximal to the origin, Supplementary Fig. S6). The plans were generated for the TO applicators used with the microSelectrion HDR. The details of the intentional errors are described in the Supplementary material. Additionally, hybrid BT plans were generated by adding 1 or 2 straight needles to evaluate the feasibility of the system for the hybrid BT plans. Plans were generated for six patients whose HR-CTV D_90%_ did not reach our goal.

### Verification of the plan data transferred to the remote afterloader

In our clinical practice, the treatment plan is transferred from the TPS to the treatment unit. The dwell time at each point was recalculated using the computer clock of the treatment unit. If errors occur in the dwell position or time during this process, many independent verification systems cannot detect them. The system developed in this study analyzes a PDF file of the pre- and post-treatment plan summaries exported over the network and compares the data with those in the DICOM RT-Plan file (Supplementary materials S1.6 and Fig. S5).

## RESULTS

**Fig. 2:**
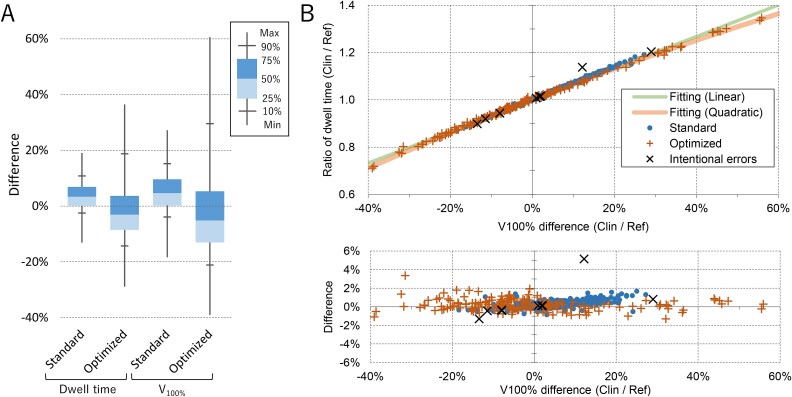
(A) Box-whisker plot of the microSelectron HDR cases: (i) the difference of the dwell time estimated by Equation 1 from the original dwell time, and (ii) the difference of the V_100%_ of Plan_Clin_ from that of Plan_Ref_. (B) Upper panel: Ratio of the dwell time of the clinical plans treated with the microSelectron HDR from that of the reference plans plotted against the difference of V_100%_. Lower panel: relative difference of the ratio shown in the upper panel from the quadratic fitting function. Plans with intentional errors are also plotted.

For the 517 plans treated with the microSelectron, the calculation time needed for one plan was 6.0 ± 1.9 s (range, 1.9–10.9 s) when using a standard computer with an Intel® Core™ i7–7700 CPU. For each microSelectron plan, T_dwell_ was estimated from Plan_Ref_ using Equation 1. The accuracy of the dwell time estimation was 3.67 ± 5.29% (range − 13.11% to 19.09%) and − 0.98 ± 12.15% (range, −28.90% to 36.48%) for the standard Manchester and optimized plans, respectively (Fig. 2). The optimized plans exhibited greater variations.

To evaluate the extent of dose distribution, V_100%_ was calculated for Plan_Ref_ and Plan_Clin_. The difference in V_100%_ from that of Plan_Ref_ showed variations similar to those of the dwell time (Fig. 2A).

In Fig. 2B, the ratio of T_dwell_ (R_dwell_: T_dwell, clin_ divided by T_dwell_ estimated by Equation 1) is plotted against the relative difference of V_100%_ (Δ_V100%_ = V_100%, clin_ / V_100%, Ref_ − 1). The lines represent the quadratic and linear fitting functions. 


(4) \begin{equation*} {\mathrm{R}}_{dwell}=0.669\bullet{\Delta}_{V_{100\%}}+1 \end{equation*}

(5) \begin{equation*} {\mathrm{R}}_{dwell}=-0.115\bullet{\Delta_{V_{100\%}}}^2+0.676\bullet{\Delta}_{V_{100\%}}+1 \end{equation*}

All plans showed a good correlation with the quadratic curve (R^2^ = 0.997), and the linear fitting also showed a good correlation (R^2^ = 0.995). The quadratic function showed better correlation, especially for points outside the ±30% range. Therefore, the T_dwell_ of Plan_Clin_ can be accurately estimated using Equation 1 with a correction of V_100%_ as follows:


(6) \begin{equation*} {T}_{dwell, clin}=\frac{D_{presc, clin}\bullet{S}_{k, ref}}{D_{presc, ref}\bullet{S}_{k, clin}}\bullet{R}_{dwell}\bullet{T}_{dwell, ref}. \end{equation*}

The bottom panel of Fig. 2B shows the relative difference in the T_dwell_ of Plan_Clin_ from the T_dwell_ estimated using Function 6 with quadratic fitting. The T_dwell_ difference between the estimation and the original plan was 0.09 ± 0.60% (range − 3.79% to 1.92%) and 0.26 ± 0.49% (range − 1.30% to 3.36%) for the linear and quadratic fitting functions, respectively. For plans with >40% V_100%_ difference, the quadratic function showed better prediction.

**Fig. 3:**
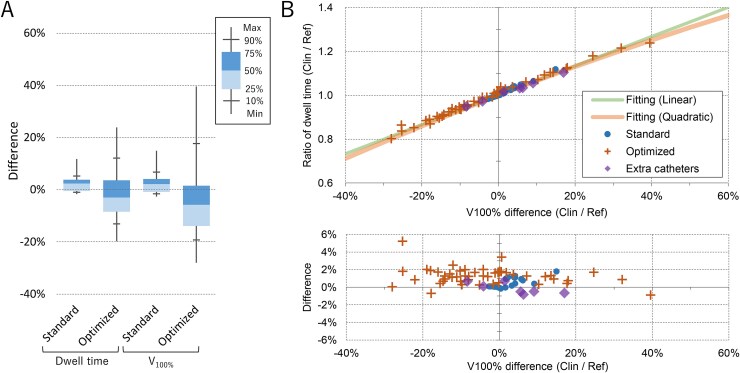
(A) Box-whisker plot of the Flexitron cases: (i) the difference of the dwell time estimated by Equation 1 from the original dwell time, and (ii) the difference of the V_100%_ of Plan_Clin_ from that of Plan_Ref_. (B) Upper panel: Ratio of the dwell time of the Plan_Clin_ treated with the Flexitron from that of Plan_Ref_ plotted against the difference of V_100%_. Lower panel: relative difference of the ratio shown in upper panel from the quadratic fitting function.

Figure 3 shows a similar analysis of the plans treated with Flexitron. The Plans_Ref_ were newly generated for the new applicators. In the top panel of Fig. 3B, the lines represent the fitting functions generated for the microSelectron HDR plans. The plots agree well with the fitting functions. The plans with additional catheters showed similar patterns. The differences for the linear and quadratic functions were 0.72 ± 0.93% (range − 2.07% to 4.08%) and 0.90 ± 0.94% (range − 0.88% to 5.24%), respectively. These data indicate that the T_dwell_ of Plan_Clin_ can be validated by estimating the dwell time using Equation 6, even for plans using different methods, including applicators, dwell positions and weight patterns.

Six hybrid BT plans using one or two additional needles were also evaluated with the Plans_Ref_ generated without needles. The difference in the T_dwell_ of Plan_Clin_ from the T_dwell_ estimated using the linear and quadratic functions were − 2.46% ± 2.81% (range − 6.79% to 1.28%) and − 1.59% ± 2.29% (range − 4.30% to 2.04%), respectively.

### Detection of human error

None of the Plan_Clin_ evaluated in this study contained human errors, including the prescribed dose, applicator length, applicator tip offset value, or dwell positions. The system successfully detected plans with intentional errors and showed warning signs. Supplementary Fig. S1 shows an example of the developed software. The parameters within tolerance are shown in green, and those with warnings are shown in orange. The T_dwell_ difference between the estimation by equation 6 and the original plan was 5.23% for the plan with irregular/unintended dwell points far from the target (Fig. 2B). For other plans with intentional errors, the T_dwell_ differences were within 1.46%.

AAPM TG-100 [24] reported a method of risk analysis based on the failure mode, and AAPM TG-275 [25] provided a list of failure modes for brachytherapy with a high score. Table 4 lists the number of failure modes detected by the system developed in this study. Four items outside the scope of the plan check and five items related to the post-treatment procedure were excluded. The proposed system developed in this study was able detected 34 items (77%). Details are presented in Supplementary Tables S1–S3.

**Table 4:** Detectability of the failure modes listed in AAPM TG-275

Category	Detectable	No	N/A	Total
Applicator placement	6	2	1	9
Imaging	6	2	1	9
Planning	22	1	3	26
Total	34 (77%)	5 (11%)	5 (11%)	44 (100%)

N/A: Not applicable to our institution or outside the scope of the verification software.

## DISCUSSION

We developed a simple model to predict T_dwell_ for ICBT in cervical cancer using a Plan_Ref_ and statistical model. Our developed software calculated the 3D dose distribution using the TG-43 formula. Instead of the *S_k_* value recorded in the DICOM RT-Plan, the value measured at our institution when exchanging the ^192^Ir source was used for the calculation and corrected for source decay. Additionally, the accuracy of the computer clock can be evaluated by checking the summary PDF file of the recalculated plan in the treatment unit. The linear fitting function is a simple equation that can accurately assess the treatment plan validity. The system enabled the accurate estimation of T_dwell_ with different applicator shapes, dwell positions and weight patterns. Additionally, the system successfully predicted T_dwell_, even for plans with extra catheters/needles, indicating that this independent verification system is useful to assess treatment adequacy when introducing new treatment machines, applicators and methods. The prediction of T_dwell_ showed large error exceeding 5% for a plan with irregular/unintended dwell points far from the target. For this plan, all DVH parameters of the target and OARs were within the clinical tolerance. Other verification systems based on point/3D dose calculations would not be able to detect this type of error. Similarly, dose constraints check of the target and OARs on the TPS would not be sufficient. We tested the method proposed by Das *et al.* [23]: the approximation formula for estimating the *Rv* value was developed for the microSelectron plans, and the *Rv* values calculated for the Flexitron plans, hybrid BT plans and plans with intentional errors were compared with the estimated values. The approximation formula accurately (within 0.01%) estimated the *Rv* values for all plans, even plans with intentional errors, indicating that the method may not detect human errors. For the Flexitron plans without any errors, the difference of T_dwell_ from the estimated value was within 3.3% for 95% of the population. Although it is difficult to determine the tolerance value, careful attention to plans with difference of estimated T_dwell_ > 3% will help to detect human errors. The estimated T_dwell_ alone cannot detect all errors, but the combination of T_dwell_ estimation and human error checking system developed in this study will improve the detectability and contribute to the rapid and effective verification of plans in clinical practice. Damato *et al.* also reported that plan verification software significantly improves the detectability of errors and efficacy of plan checks [26].

Many studies have investigated the plan verification methods for brachytherapy [12–14, 27]. Some plan-checking systems evaluate plan validity by calculating the DVH of the target and OARs [14, 28]. A key feature of ICBT for cervical cancer is that delineation of the target contour is not essential for the classical Manchester method. DVH-based verification systems may not be effective at detecting errors around the tandem tip if the entire uterus is not delineated. Takahashi *et al.* reported a case of TC treated with an incorrect tandem length protocol, resulting in inappropriate dwell positions [22]. This error would not have been detected by DVH-based verification if the vaginal CTV had not been delineated.

Currently some TPSs have a library planning function, which uses preset of dwell positions and weights. If the type of applicator used (e.g. tandem length or cylinder diameter) is different from the plan document, the planner may load a wrong plan library. This type of error cannot be detected by plan checking systems that evaluate dose calculation accuracy (e.g. 3D gamma or DVH). Our system will detect the error even if incorrect Plan_Ref_ is selected because the system also checks the geometry, such as the distance between the most distal/proximal dwell point and the origin. For recent TPSs, applicator library modeling, which allows semi-automatic applicator reconstruction based on the applicator library with 3D geometry. This function will greatly reduce such human errors.

The software successfully detected 77% of the failure modes listed in AAPM TG-275 with a high score [25]. Many of the remaining failure modes were outside the scope of the verification software (i.e. infection, perforation and unlocked applicator) or techniques not used at our institution (i.e. biologically effective dose, image registration with MRI and heterogeneity corrections).

Cai *et al.* [28] and Simiele *et al.* [29] reported verification systems using scripting with an application programming interface (API). The API-based system can quickly analyze plan quality without exporting plan data in DICOM format. However, verification systems integrated with TPS may not be able to detect errors related to the recalculation of the dwell time with the remote afterloader’s computer clock. The system developed in this study checks the following three temporal parameters and notifies users when the parameters are out of tolerance: (i) the time elapsed from the CT image acquisition; (ii) the time difference between the treatment time recorded in the DICOM RT-Plan and the time at the plan check prior to the treatment and (iii) the difference between the dwell times and positions recorded in the pre-treatment plan summary PDF file exported from the remote afterloader and the values recorded in the DICOM RT-Plan. The number of dwell positions in Flexitron is much higher than that in older machines because the minimum step size of the dwell positions is 1 mm. An automatic check of all dwell positions and dwell times will contribute to a quick and effective plan check.

This study has some limitations. This system successfully worked for hybrid BT plans with additional needles. However, it cannot estimate interstitial brachytherapy (ISBT) plans generated only with needles, including ISBT for bulky or recurrent gynecological cancer and also for rectal, prostate and head and neck cancer, because Plans_Ref_ cannot be defined. The other check functions work for many parameters, including applicator length, offsets, imaging, source strength and DVH. Because the dwell time of the DICOM RT-Plan can be scaled up with correction for source decay, the pre- and post-treatment summaries exported from the treatment unit can be evaluated not only for the initial treatment, but also for all treatment fractions. This system is applicable to treatments whose reference patterns can be defined, such as accelerated partial breast irradiation. Additionally, we tested only Elekta afterloaders with a ^192^Ir source. Other vendors and sources such as ^60^Co require further investigation.

We developed a simple independent verification method and a system that automatically detected errors in ICBT treatment planning for cervical cancer. The newly developed approximation formula accurately estimated the T_dwell_, even for plans created using different remote after loader, applicators and treatment methods. Other verification algorithms including Plan_Ref_-based geometry checks, imaging, source decay and pre- and post-treatment report checks will be helpful in reducing human errors and system disfunctions that cannot be detected by dosimetry-based verification systems. The integrated system would contribute to the safety of ICBT when introducing new treatment modalities and methods.

## Supplementary Material

Supplementary_material_rraf007

## FINDING

This work was supported by JSPS KAKENHI Grant Number JP20K08022 and JP23K07109.

## PRESENTATION AT A CONFERENCE

This work was presented at the 26th Annual Meeting of the Japanese Group of Brachytherapy/Japanese Society for Radiation Oncology.
