# Supplementary material for: Independent verification system for intracavitary brachytherapy based on a reference plan and statistical model
Source: J Radiat Res. 2025 Mar 5;66(2):176–84. doi: 10.1093/jrr/rraf007 (PMC11932347; doi:10.1093/jrr/rraf007)
Supplement: Supplementary_material_rraf007 [file supplementary_material_rraf007.pdf]

Supplementary material

S1. Parameters checked by the verification software.

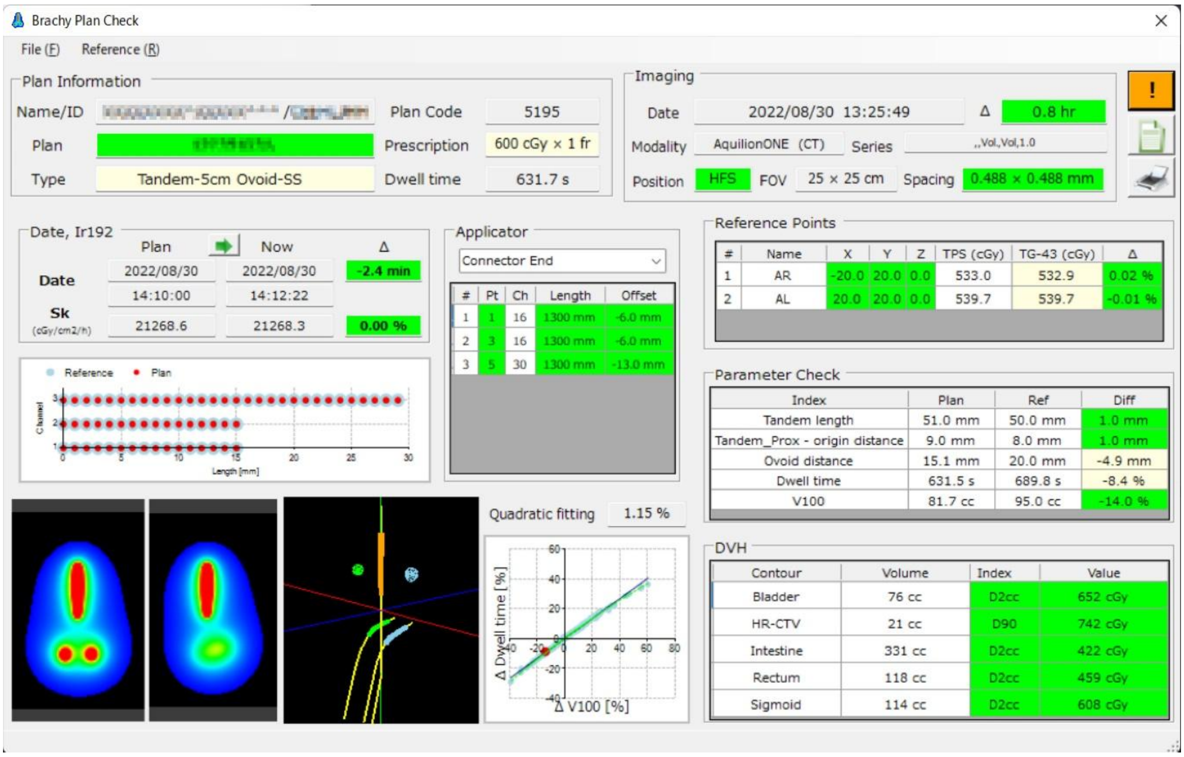

Figure S1. An example case analyzed with the verification software.

S1.1. Image data

DICOM CT files with the same Frame of Reference UID to that of the DICOM RT-Plan file were automatically found. The acquisition date/time of the CT files was compared with the current computing time. The software also checks whether the patient's orientation is head-first. The field of view (FOV) and image resolution were also evaluated.

S1.2. Date/time and S_k

The software compares the current date/time of the computer clock with the treatment date/time recorded in the DICOM RT-Plan file. In addition, the air-kerma source strength (S_k) was calculated from the date/time and S_k of the ^{192}Ir source measured at our institution when the source was exchanged and the elapsed time. The S_k value recorded in the RT-Plan file was compared with the calculated value.

S1.3. Applicators and source positions

The software compared the following parameters with those of $Plan_{Ref}$: channel mapping, applicator length, offset values of the first dwell position from the applicator tip, patterns of the dwell positions, step size between each dwell position, and weight of each dwell point. The offset from the applicator tip was calculated along the 3D vector of the first dwell point. The offsets were calculated from the 3D coordinates of the applicator contour, which were determined manually from the CT image during planning. When the "connector-end" is selected as the applicator digitization mode, the offset is calculated as the first dwell position subtracted by the last point of the applicator contour. If the "tip-end" mode is selected during planning, exceptionally large offset values are observed.

The dwell positions of $Plan_{Clin}$ and $Plan_{Ref}$ are shown in Figure S2. The large blue and small red points represent the dwell positions in the reference and clinical plans, respectively. The points were plotted against the distance from the first dwell position. The exact overlap of these point sets indicates that the correct planning patterns were loaded from the library.

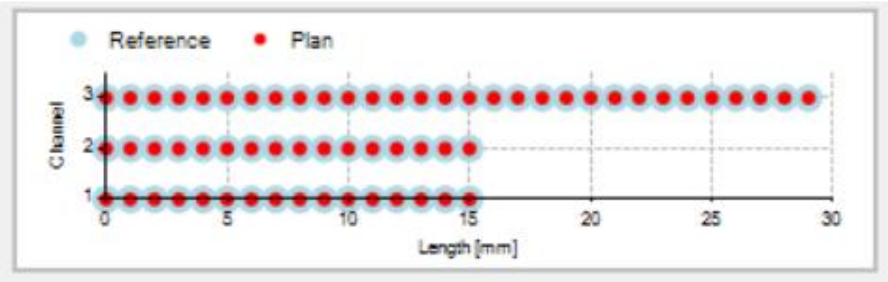

Figure S2. Dwell positions of clinical and reference plans.

S1.4. Geometric parameters

Geometric parameters were defined to check for incorrect construction of the applicators and incorrect selection of the plan template from the predefined plan library. Tandem length was calculated for the tandem and ovoid (TO) and tandem cylinder (TC) plans. The ranges of the dwell positions in the cylinder were calculated for the TC and cylinder plans. For the TO plans, the distance from the most proximal dwell position of the tandem to the origin and the distance between the two ovoid applicators were also calculated. For the cylinder plans, the cylinder diameter was calculated from the coordinates of two reference points located 5 mm from the cylinder surface.

The applicators and reference points are visualized as 3D images (Fig. S3). An incorrect applicator placement and digitization were performed. For the TO plans, the colors of channels 1 (ovoid of patient right) and 2 (ovoid of patient left) were green and blue, respectively. Point A with negative (patient right) and positive (patient left) coordinates on

the horizontal axis are colored green and blue, respectively. Therefore, the colors of the ovoid and Point-A must be the same for both the right and left sides. Incorrect channel mapping can easily be found.

The DICOM RT-structure file was automatically identified by checking the Referenced SOP instance UID recorded in the DICOM RT-Plan file. The OAR contours are shown in a 3D graphic (Fig. S4). The color of each contour is automatically selected by the contour name, but is independent of the color recorded in the DICOM RT-Structure file. If the contours were delineated by an incorrect structure, the reviewer would find the view.

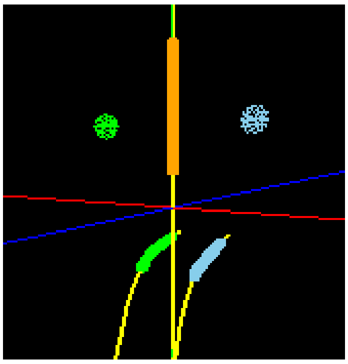

Figure S3. A 3D graphic view of the applicators.

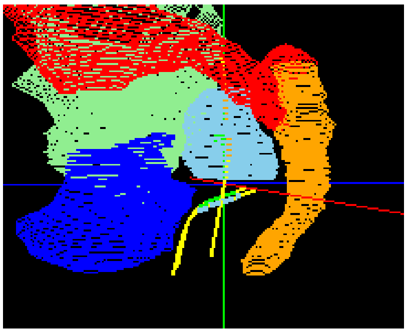

Figure S4. 3D graphic view showing structure.

S1.5. Dosimetric evaluations

If the prescribed dose of the $Plan_{Clin}$ is different from that of $Plan_{Ref}$, the software shows a notification or warning depending on the difference. This software calculates the dose using the dwell time at each point recorded in the DICOM RT-Plan file and the S_k registered to the verification software considering the decay. A line-source model of the AAPM TG-43 formula was used in this study.

The number of reference points and the coordinates of the points were compared with those in $\mathrm{Plan}_{\mathrm{Ref}}$. The doses at these points were calculated and compared with values recorded in the DICOM and Communications in the RT-Plan File. The doses at the reference points were also compared with those in $\mathrm{Plan}_{\mathrm{Ref}}$, considering the change in the prescribed dose. If $\mathrm{Plan}_{\mathrm{Clin}}$ is not optimized, a small difference between $\mathrm{Plan}_{\mathrm{Clin}}$ and $\mathrm{Plan}_{\mathrm{Ref}}$ is expected.

In addition, the 3D dose distribution was calculated inside all target and OAR contours and for voxels located within 3 cm of the nearest dwell point to calculate the volume receiving 100% of the prescribed dose ($V_{100\%}$). The total dwell time was estimated using $V_{100\%}$ and Equation 4 in the manuscript. The $D_{90\%}$ of the HR-CTV and $D_{2\,cc}$ of the OARs were also evaluated to determine whether the DVH parameters of the target and OARs were within the tolerance range.

S1.6. Pre-, Post-treatment reports

After treatment planning, the plan was transferred from the TPS to the treatment unit. The dwell time at each point was re-calculated with the computer time for the treatment unit. Before and after treatment, PDF files were exported via the network, including patient ID, plan name, plan code, channel number, catheter length, dwell time, and positions. These values were compared with those of the DICOM RT-Plan file (Fig. S5).

Here, the dwell time of the DICOM RT-Plan file can be scaled up by considering source decay. Therefore, the dwell time of all positions recorded in the pretreatment summary exported by the treatment unit can be checked not only for the initial treatment but also for all treatment fractions of interstitial brachytherapy, which uses the same plan for multiple fractions.

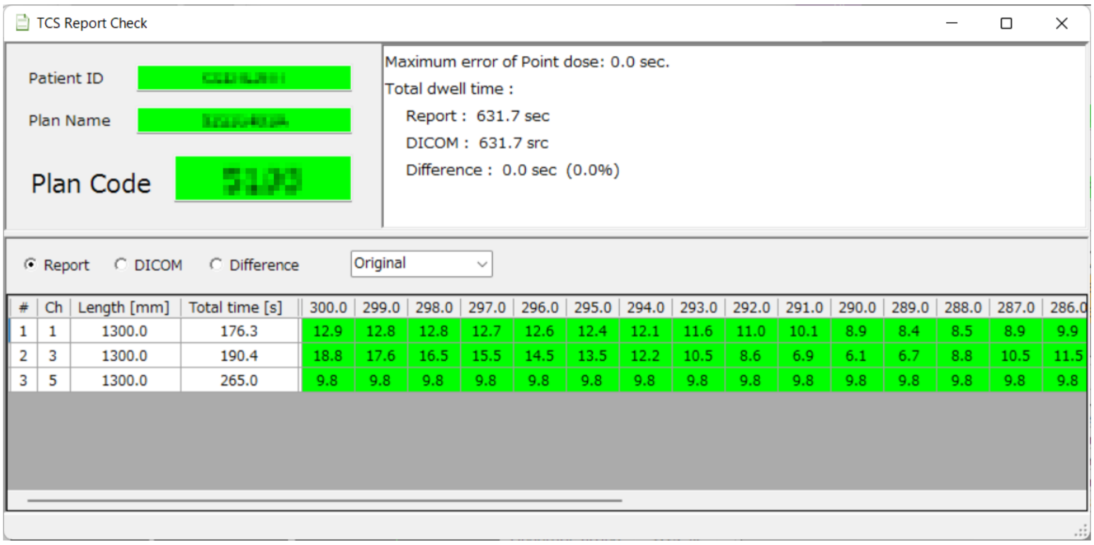

Figure S5. Checking the pre- and post-treatment summary exported from the treatment unit.

S2.Details of the plans with intentional errors

(i) Incorrect applicator channel mapping in TO plans

Correct channel (Ch) mapping is as follows: Ch 1 for right ovoid, Ch 2 for left ovoid, and Ch 3 for tandem. Two plans with intentional errors were generated: (A) Opposite ovoid channels: Ch1 for left ovoid and Ch2 for right ovoid; and (B) incorrect tandem channel: Ch1 for tandem, Ch2 for right ovoid, and Ch3 for left ovoid.

(ii) Incorrect offset values for the first dwell position from the applicator tip

The correct offset value is -7 mm for all ovoid and tandem applicators. Two plans with intentional errors were generated: (C) the offset of Ch1 is zero, but the values of the others are correct; (D)the offset of all applicators are +7mm.

(iii) Selection of the incorrect plan template from the library

The applicators were constructed using the 6-cm long tandem applicator. Two plans with intentional errors were generated: plans generated using the plan library of (E) 5-cm long and (F) 7-cm long tandem applicators. Therefore, the number of dwell points and the weights of the dwell positions along the tandem applicators are different from those of the reference plan of 6-cm long tandem applicator.

(iv) Identification of the applicator coordinates with incorrect digitization mode

During the process of the catheter reconstruction using “connector end” mode, the applicator geometry should be identified from the connector side (proximal) to the tip (distal) direction. For the plan with the intentional error (G), the applicators were identified with this direction but using the “tip end” mode.

(v) Incorrect reference points

The reference points (Point A) should be placed at two positions (2 cm superior and 2 cm bilateral). For the plan with intentional error (H), third reference point was placed near the right ovoid tip, assuming the planner’s unintentional operation. The dose distribution is rescaled by normalizing the dose at these three points to the prescribed dose.

(vi) Irregular/unintended dwell points far from the target

For the plan with intentional error (I), a dwell point with a dwell time of 40 s was added on the tandem at 5 cm proximal to the origin (Fig. S6).

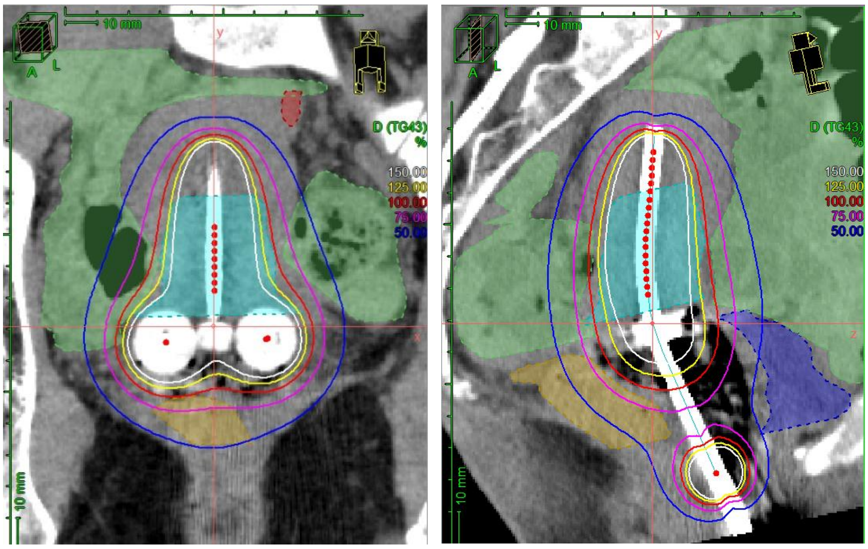

Figure S6. Dose distribution of the (vi) irregular/unintended dwell points far from the target. Coronal (left) and Sagittal (right) images are shown.

Table S1. Detectability of the failure modes related to applicator placement.

Failure mode	Detectability	Comments
Insufficient packing around applicator	Detectable	DVH
Ovoid crossed	Detectable	Geometric analysis
Ring not inserted higher enough	Detectable	Geometric analysis
Angled tandem inserted upside down	Detectable	
Mismatch of tandem/ovoid angle	Detectable	3D Graphic
Catheter kinked	Detectable	3D Graphic
Perforation	No	
Incorrect applicator inserted	No	
Shielded cylinder	N/A	Not used
Cylinder inserted into wrong orifice	O/S	
Infection	O/S	
Applicator not locked	O/S	
Flange not flush	O/S	

Abbreviations: N/A, not applicable to our institution; O/S, outside the scope of the verification software (not included in Table 4 of the manuscript); DVH, dose volume histogram.

Table S2. Detectability of the failure modes related to imaging.

Failure mode	Detectability	Comments
Incorrect channel length and number	Detectable	
Wrong dataset exported	Detectable	Time elapsed from image acquisition
Incorrect scan orientation	Detectable	
Incorrect scan parameters	Detectable	
Incorrect bladder fill	Detectable	DVH
Insufficient FOV of imaging	Detectable	3D Graphic
Wrong marker insertion	No	
Patient not eligibility for imaging	No	
Compatibility of the applicators for Imaging	N/A	MRI is not used

Abbreviations: N/A, not applicable to our institution; FOV, field of view; MRI, magnetic resonance imaging.

Table S3. Detectability of the failure modes related to planning.

Failure mode	Detectability	Comments
Incorrect catheter digitization	Detectable	
Incorrect treatment length	Detectable	
Incorrect distal reference length	Detectable	
Incorrect selection of applicator	Detectable	
Incorrect applicator model placement	Detectable	
Transfer of different plan	Detectable	Checking approval and plan time
Dwell time not updated	Detectable	
DVH constraints not met	Detectable	
Incorrect selection of connector end vs tip end	Detectable	
Wrong prescribed dose and fraction	Detectable	
Guidelines not met	Detectable	DVH
Incorrect plan normalization	Detectable	
Protocol not met	Detectable	
Wrong dataset	Detectable	
Wrong step size	Detectable	
Wrong selection of source	Detectable	
Wrong selection of device	Detectable	
Secondary dose calculation not done	Detectable	
Incorrect channel mapping	Detectable	
Wrong contouring	Detectable	3D Graphic
Dwell position outside applicator	Detectable	3D Graphic
Incorrect labeling of OAR	Detectable	3D Graphic
Prior treatment not considered	No	
Incorrect BED calculation	N/A	Not used
Poor image registration	N/A	Not used
Incorrect heterogeneity corrections	N/A	Not used

Abbreviations: N/A, not applicable to our institution; DVH, dose-volume-histogram; OAR, organs at risk; BED, biological effective dose.
